# Enhanced passive safety surveillance of three marketed influenza vaccines in the UK and the Republic of Ireland during the 2017/18 season

**DOI:** 10.1080/21645515.2019.1581538

**Published:** 2019-03-27

**Authors:** Sonja Gandhi-Banga, Anne-Laure Chabanon, Cecile Eymin, Timothy Caroe, Karina Butler, Annick Moureau

**Affiliations:** a Sanofi Pasteur, Toronto, ON, Canada; b Sanofi Pasteur, Lyon, France; c Lighthouse Medical Practice, Eastbourne, East Sussex, UK; d Our Lady’s Children’s Hospital, Dublin, Crumlin, Ireland; e Sanofi Pasteur, Marcy l’Etoile, France

**Keywords:** Influenza vaccines, vaccination, safety, safety surveillance, seasonal influenza, adverse reaction, adverse event, reactogenicity, immunization

## Abstract

Safety surveillance is required for each season’s influenza vaccines to rapidly detect and evaluate potential new safety concerns before the peak period of immunization. Here we report the results of an enhanced passive safety surveillance for a trivalent split-virion inactivated influenza vaccine (IIV3; Vaxigrip®), an intradermal version of this vaccine (IIV3-ID; Intanza® 15 µg), and a recently licensed quadrivalent version (IIV4; VaxigripTetra^TM^) during the 2017/18 influenza season in the UK and Republic of Ireland. The primary objective was to determine the rates of adverse reactions (ARs) occurring within 7 days following routine vaccination. Between September and November 2017, 979 safety report cards were distributed to vaccinees receiving IIV3-ID, 1005 to those receiving IIV3, and 957 to those receiving IIV4. At least one AR was reported by 28 participants (2.9%) vaccinated with IIV3-ID, 14 participants (1.4%) vaccinated with IIV3, and 20 participants (2.1%) vaccinated with IIV4. The most frequent ARs were injection-site reactions and headache. One participant vaccinated with IIV3-ID reported two suspected serious ARs (dyskinesia and a shock symptom), although these could not be confirmed as vaccine-related. Rates of ARs for IIV3 and IIV3-ID for 2017/18 did not differ from the 2016/17 rates. For IIV4, in its first season since licensure, AR frequencies were similar to those in the Summary of Product Characteristics. In conclusion, no change was found compared to the known or expected AR rates for IIV3, IIV3-ID, or IIV4 during the 2017/18 season.

## Introduction

Seasonal influenza vaccination is recommended to older adults and other at-risk populations in many countries.^1^ The vaccine strains are often changed from season to season in response to antigenic changes in circulating viruses. Since 2014, the European Medicines Agency (EMA) has required enhanced safety surveillance for all seasonal influenza vaccines, and specific guidance has been produced by the EMA’s Pharmacovigilance Risk Assessment Committee (PRAC).^2^ This surveillance is intended to rapidly detect and evaluate potential new safety concerns before the peak period of immunization. Safety concerns may be signaled by increased rates of reactogenicity or allergic events compared to those expected or measured from the previous season’s vaccine. Increases in these rates might indicate a potential for more serious risks as exposure to the vaccine increases.^2^

Enhanced passive safety surveillance (EPSS) is a type of enhanced surveillance design that is encouraged by the EMA.^2^ EPSS increases adverse event reporting by combining passive surveillance with clinical services that encourage patients and healthcare professionals to report adverse events.^3^ These improvements have been shown to reduce the under-reporting of adverse events that often occurs in routine pharmacovigilance systems.^3–5^

An intramuscularly administered trivalent split-virion inactivated influenza vaccine (IIV3; Vaxigrip®, Sanofi Pasteur) has been available since 1968 for individuals aged 6 months and older.^6^ An intradermally administered version (IIV3-ID; Intanza® 15 µg, Sanofi Pasteur) was licensed in 2009 and is indicated for individuals aged 60 years and older.^7^ The results of EPSS have been previously published for these two vaccines during the Northern Hemisphere (NH) 2016/17 and 2015/16 influenza seasons.^8,9^

Most recently, an intramuscularly administered quadrivalent split-virion influenza vaccine (IIV4; VaxigripTetra^TM^, Sanofi Pasteur) was licensed in Europe in 2016 for individuals aged 3 years and older.^10^ Its indication has since been extended to children from 6 months of age in many countries. Because it has only recently been licensed, IIV4 was not included in previous EPSS studies.^8,9^ Here, we describe the EPSS results for these three vaccines during the NH 2017/18 influenza season and compare them with the NH 2016/17 season rates for IIV3 and IIV3-ID and with reference safety information for IIV4.

## Results

### Exposure data

Between September 20 and November 3, 2017, 12 healthcare professionals in the UK and one healthcare professional in the Republic of Ireland distributed 979 safety report cards for IIV3-ID, 1005 for IIV3, and 957 for IIV4 (Table 1). Because of errors made during distribution, the target of 1000 safety report cards was not met for IIV3-ID and IIV4. The EPSS covered one batch of IIV3-ID, nine batches of IIV3, and eight batches of IIV4. Almost all participants (98.2%) vaccinated with IIV3 or IIV4 were aged ≥18 years and all participants vaccinated with IIV3-ID were aged ≥60 years, consistent with its indication.

**Table 1.** Overall frequencies of suspected adverse reactions (ARs) and adverse events of interest (AEIs) occurring within 7 days by vaccine and age group.

		Participants reporting ≥1 AR		#ARs		Participants reporting ≥1 AEI^a^		#AEIs^a^	
	Safety report cards distributed	n	% (95% CI)	n	%	n	% (95% CI)	n	%
**IIV3-ID**									
≥60 y	977	27	2.8 (1.7, 3.8)	71	7.3	21	2.1 (1.2, 3.1)	36	3.7
Total	979^b^	27	2.8 (1.7, 3.8)	71	7.3	21	2.1 (1.2, 3.1)	36	3.7
**IIV3**									
6 mo−5 y	7	0	0.0 (−)	0	0.0	0	0.0 (−)	0	0.0
6−12 y	13	0	0.0 (−)	0	0.0	0	0.0 (−)	0	0.0
13−17 y	6	0	0.0 (−)	0	0.0	0	0.0 (−)	0	0.0
18−65 y	326	5	1.5 (0.5, 3.5)	9	2.8	4	1.2 (0.3, 3.1)	6	1.8
>65 y	653	8	1.2 (0.4, 2.1)	21	3.2	5	0.8 (0.3, 1.8)	7	1.1
Total	1005	13	1.3 (0.6, 2.0)	30	3.0	9	0.9 (0.3, 1.5)	13	1.3
**IIV4**									
3−5 y	0	0	0.0 (−)	0	0.0	0	0.0 (−)	0	0.0
6−12 y	4	0	0.0 (−)	0	0.0	0	0.0 (−)	0	0.0
13−17 y	6	0	0.0 (−)	0	0.0	0	0.0 (−)	0	0.0
18−65 y	276	5	1.8 (0.6, 4.2)	12	4.3	4	1.4 (0.4, 3.7)	7	2.5
>65 y	671	13	1.9 (0.9, 3.0)	27	4.0	10	1.5 (0.6, 2.4)	12	1.8
Total	957	18	1.9 (1.0, 2.7)	39	4.1	14	1.5 (0.7, 2.2)	19	2.0

Abbreviations: IIV3, trivalent split-virion inactivated influenza vaccine; IIV3-ID, intradermally administered trivalent split-virion inactivated influenza vaccine; IIV4, quadrivalent split-virion inactivated influenza vaccine; −, not reported

^a^ AEIs were defined according to the Pharmacovigilance Risk Assessment Committee^2^

^b^ Two participants had ages outside the indicated age range; neither reported an AR during the surveillance period

### Safety data

#### IIV3-ID

Seventy-seven ARs were reported by 28 participants (2.9%) vaccinated with IIV3-ID over the EPSS period, most of which were reported within 7 days of vaccination (Tables 1 and 2). The reported ARs included 37 adverse events of interest (AEIs), the most frequent of which were at the vaccination site (inflammation, pruritus, reaction, and erythema). Most AEIs were mild or moderate in severity (Table S1). The most frequent suspected ARs not considered AEIs were oropharyngeal pain and hyperhidrosis.

**Table 2.** Frequencies of all adverse reactions (ARs) for IIV3-ID and IIV3 reported during the 2017/18 EPSS and comparison with the frequencies reported during the 2016/17 EPSS.

ARs	IIV3-ID				IIV3			
	2017/18 (N = 979)		2016/17 (N = 1000)		2017/18 (N = 1005)		2016/17 (N = 962)	
	n	% (95% CI)	n	% (95% CI)	n	% (95% CI)	n	% (95% CI)
Participants reporting ≥1 suspected AR	28	2.9 (1.8, 3.9)	21	2.1 (1.2, 3.0)	14	1.4 (0.7, 2.1)	17	1.8 (0.9, 2.6)
Participants reporting ≥1 AEI	22	2.2 (1.3, 3.2)	17	1.7 (0.9, 2.5)	10	1.0 (0.4, 1.6)	12	1.2 (0.5, 1.9)
Suspected ARs^a^	77	7.9	103	10.3	40	4.0	59	6.1
AEIs^a, b^	37	3.8	44	4.4	17	1.7	28	2.9
Headache	1	0.1 (0.0, 0.6)	6	0.6 (0.1, 1.1)	3	0.3 (0.1, 0.9)	4	0.4 (0.1, 1.0)
Feeling of body temperature change	2	0.2 (0.0, 0.7)	0	0.0 (0.0, 0.4)	−	−	−	−
Malaise	1	0.1 (0.0, 0.6)	6	0.6 (0.1, 1.1)	2	0.2 (0.0, 0.7)	4	0.4 (0.1, 1.0)
Nausea	−	−	−	−	2	0.2 (0.0, 0.7)	0	0.0 (0.0, 0.4)
Vaccination site erythema	3	0.3 (0.1, 0.9)	6	0.6 (0.1, 1.1)	−	−	−	−
Vaccination site inflammation	10	1.0 (0.4, 1.7)	3	0.3 (0.1, 0.9)	6	0.6 (0.1, 1.1)	0	0.0 (0.0, 0.4)
Vaccination site pruritus	6	0.6 (0.1, 1.1)	2	0.2 (0.0, 0.7)	−	−	−	−
Vaccination site reaction	3	0.3 (0.1, 0.9)	1	0.1 (0.0, 0.6)	−	−	−	−
Other ARs^b^								
Asthenopia	2	0.2 (0.0, 0.7)	0	0.0 (0.0, 0.4)	−	−	−	−
Inflammation	2	0.2 (0.0, 0.7)	0	0.0 (0.0, 0.4)	−	−	−	−
Lethargy	1	0.1 (0.0, 0.6)	0	0.0 (0.0, 0.4)	2	0.2 (0.0, 0.7)	1	0.1 (0.0, 0.6)
Nasopharyngitis	2	0.2 (0.0, 0.7)	1	0.1 (0.0, 0.6)	−	−	−	−
Oropharyngeal pain	3	0.3 (0.1, 0.9)	8	0.8 (0.2, 1.4)	2	0.2 (0.0, 0.7)	1	0.1 (0.0, 0.6)
Rhinorrhea	2	0.2 (0.0, 0.7)	4	0.4 (0.1, 1.0)	−	−	−	−
Hyperhidrosis	3	0.3 (0.1, 0.9)	0	0.0 (0.0, 0.4)	2	0.2 (0.0, 0.7)	0	0.0 (0.0, 0.4)

Abbreviations: AEI, adverse event of interest; CI, confidence interval; IIV3, trivalent split-virion inactivated influenza vaccine; IIV3-ID, intradermally administered trivalent split-virion inactivated influenza vaccine; −, none reported

^a^ Since the proportion of total reported ARs and AEIs could theoretically be >100%, 95% CIs were not calculated

^b^ Only ARs reported in ≥2 participants in NH Season 2017/18 are shown

Two suspected serious ARs (dyskinesia and a shock symptom) were reported by a 74-year-old female participant. She also experienced rhinorrhea, eye pain, asthenopia, dysuria, feeling of body temperature change, ocular hyperemia, feeling cold, feeling abnormal, and limb discomfort. All of these events were considered non-serious ARs. Although referred to her physician, the participant did not seek consultation for these events, no diagnosis could be made, and a causal relationship with IIV3-ID could not be excluded. The participant was reported to be recovering from the ARs during the last contact made.

Overall, AR and AEI rates for IIV3-ID during the 2017/18 season were similar to those reported in the previous influenza season (Table 2).

#### IIV3

Forty ARs were reported by 14 participants (1.4%) vaccinated with IIV3 (Table 2). All ARs with known onset (n = 30) occurred within 7 days of vaccination (Table 1). The reported ARs included 17 AEIs, the most frequent of which were inflammation at the vaccination site and headache (Table 2). The most frequent suspected ARs not considered AEIs were lethargy, oropharyngeal pain, and hyperhidrosis. No serious ARs were reported. No ARs were reported in participants aged from 6 months to 17 years.

AR and AEI rates for IIV3 during the 2017/18 season were comparable to those reported during the previous influenza season (Table 2).

#### IIV4

Fifty-six ARs were reported by 20 subjects (2.1%) vaccinated with IIV4 (Table 3). All ARs with known onset (n = 39) occurred within 7 days of vaccination (Table 1). The reported ARs included 25 AEIs, the most frequent of which were headache, fever, and vaccination-site inflammation (Table 3). The most frequent suspected ARs not considered AEIs were fatigue and oropharyngeal pain. No serious ARs were reported after vaccination with IIV4. No ARs were reported in the ten participants aged 3 to 17 years.

**Table 3.** Frequencies of all adverse reactions (ARs) reported for IIV4 during the 2017/18 EPSS and comparison with the frequency categories listed in the SmPC.

		18–65 y (N = 276)				>65 y (N = 671)		
	2017/18				2017/18			
	n	% (95% CI)	Category	SmPC^d^ category	n	% (95% CI)	Category	SmPC^d^ category
Participants reporting ≥1 suspected AR	5	1.8 (0.6, 4.2)			15	2.2 (1.1, 3.4)		
Participants reporting ≥1 AEI	4	1.4 (0.4, 3.7)			12	1.8 (0.8, 2.8)		
Suspected ARs^a^	13	4.7			43	6.4		
AEIs^a, b^	7	2.5			18	2.7		
Fever^c^	1	0.4 (0.0, 2.0)	Uncommon	Common	2	0.3 (0.0, 1.1)	Uncommon	Uncommon
Headache	2	0.7 (0.1, 2.6)	Uncommon	Very common	5	0.7 (0.2, 1.7)	Uncommon	Very common
Vaccination site inflammation	1	0.4 (0.0, 2.0)	Uncommon	Mixed^e^	2	0.3 (0.0, 1.1)	Uncommon	Mixed^e^
Malaise	1	0.4 (0.0, 2.0)	Uncommon	Very common	1	0.1 (0.0, 0.8)	Uncommon	Common
Myalgia	0	0.0 (−)	−	Very common	2	0.3 (0.0, 1.1)	Uncommon	Very common
Other ARs^b^								
Fatigue	1	0.4 (0.0, 2.0)	Uncommon	Uncommon	2	0.3 (0.0, 1.1)	Uncommon	Uncommon
Feeling hot	0	0.0 (−)	−	−	2	0.3 (0.0, 1.1)	Uncommon	−
Pain	1	0.4 (0.0, 2.0)	Uncommon	−	1	0.1 (0.0, 0.8)	Uncommon	−
Nasopharyngitis	0	0.0 (−)	−	−	2	0.3 (0.0, 1.1)	Uncommon	−
Pain in extremity	1	0.4 (0.0, 2.0)	Uncommon	−	1	0.1 (0.0, 0.8)	Uncommon	−
Dizziness	0	0.0 (−)	−	Rare	2	0.3 (0.0, 1.1)	Uncommon	Uncommon
Lethargy	0	0.0 (−)	−	Rare	2	0.3 (0.0, 1.1)	Uncommon	Rare
Oropharyngeal pain	0	0.0 (−)	−	−	3	0.4 (0.1, 1.3)	Uncommon	−
Rhinorrhea	0	0.0 (−)	−	−	2	0.3 (0.0, 1.1)	Uncommon	−

Abbreviations: AEI, adverse event of interest; CI, confidence interval; IIV4, quadrivalent split-virion inactivated influenza vaccine; PRAC, Pharmacovigilance Risk Assessment Committee; SmPC, Summary of Product Characteristics; −, not reported

^a^ Since the proportion of total reported ARs and AEIs could theoretically be >100%, 95% CIs were not calculated

^b^ Only ARs reported in ≥2 participants in NH Season 2017/18 are shown

^c^ Includes feeling of body temperature change or pyrexia

^d^ Frequencies: very common (≥10%); common (≥1% to <10%); uncommon (≥0.1% to <1%); rare (≥0.01% to <0.1%); very rare (<0.01%)

^e^ Pain (very common; ≥10%), erythema and swelling (common; ≥1% to <10%), warmth (uncommon; ≥0.1% to <1%)

The frequency categories determined from the reported ARs did not differ from those documented in the Summary of Product Characteristics (SmPC) for IIV4, except that for participants aged ≥65 years, lethargy was one frequency category higher during 2017/18 (“uncommon”) than the corresponding AR term documented in the SmPC (“rare”) (Table 3).

## Discussion

Early influenza vaccine safety surveillance aims to identify potential new safety concerns as soon as possible, before the peak period of mass immunization.^2^ As in previous EPSS studies,^8,9^ no safety issues were observed during the NH 2017/18 influenza season in participants receiving IIV3-ID or IIV3. Similarly, for IIV4, the EPSS showed no difference between the reported rates of ARs and those expected based on clinical trial data. The most common ARs for the three vaccines were mild and transient injection-site reactions, headache, and fever, which are frequently reported for these and other influenza vaccines.^6,10,11^ As reported elsewhere,^11^ injection-site reactions were more frequent among participants receiving IIV3-ID, although the reported rate was lower than that listed in the SmPC.^12^ An increased frequency of injection-site reactions is expected for intradermal vaccination compared to intramuscular injection,^13^ probably due to higher, more localized antigen delivery and higher concentrations of immune cells in the skin.^14^

One participant reported two serious suspected ARs, dyskinesia and a shock symptom, after vaccination with IIV3-ID. The reported symptoms were not consistent with a diagnosis of anaphylactic or anaphylactoid shock, and therefore are not considered severe allergic reactions. A causal relationship between these serious ARs and the vaccine could not be confirmed.

This EPSS was based on spontaneous, near real-time reporting using safety report cards. This study design, reinforced by communicating to participants the importance of reporting ARs, can increase reporting rates in passive surveillance systems.^15^ Nevertheless, under- or differential reporting can still occur because reporting remains spontaneous, as noted in similar surveillance system designs.^3,16–18^ A further limitation was that slightly fewer than the targeted 1000 safety report cards were distributed to participants receiving IIV3-ID and IIV4, although the small difference was unlikely to affect the conclusions. However, children and adolescents receiving IIV3 and IIV4 were poorly represented, possibly due to several sites overestimating their potential to vaccinate younger age groups within the short surveillance period. An additional factor could have been that intranasally administered live attenuated influenza vaccine is recommended for children in the UK.^19^ A final limitation was that only one batch of IIV3-ID could be included, because no further batches were released in the UK during the surveillance period.

Overall, the EPSS results suggested no clinically significant change in the safety of the three vaccines for the NH 2017/18 season. In addition, the 2017/18 EPSS provides some of the first real-world post-marketing safety data for IIV4, from its first season in routine clinical practice. These results are important for encouraging influenza vaccination among healthcare professionals and persons at greater risk of influenza complications.

## Patients and methods

### Study design

This was a multicenter, non-interventional EPSS conducted between September and November 2017 in the UK and Republic of Ireland. The primary objective was to determine the rates of suspected ARs occurring within 7 days following routine vaccination with IIV3 (Vaxigrip, also called Inactivated Influenza Vaccine [split virion] BP; Sanofi Pasteur), IIV3-ID (Intanza 15 µg; Sanofi Pasteur), or IIV4 (VaxigripTetra, also called Quadrivalent influenza vaccine [split virion, inactivated]; Sanofi Pasteur) during the NH 2017/18 influenza season. Secondary objectives were to estimate rates of suspected ARs by age group; estimate rates of serious suspected ARs; and compare the rates of suspected ARs with those recorded in the previous 2016/17 NH influenza season or, for IIV4, with the AR frequencies documented in the SmPC. The study was approved by the local independent ethics committees and conducted in accordance with the Declaration of Helsinki, Good Epidemiological Practice, and the European Network of Centres for Pharmacoepidemiology and Pharmacovigilance. Informed consent was not required because the EPSS relied on routine pharmacovigilance and voluntary spontaneous reporting.

### Vaccine formulations

The 2017/18 vaccine strains used for IIV3 and IIV3-ID were A/Michigan/45/2015 (H1N1)pdm09, A/Hong Kong/4801/2014 (H3N2), and B/Brisbane/60/2008. IIV4 included these three strains plus the additional B lineage strain B/Phuket/3073/2013.

### Study conduct and data collection

Healthcare professionals were selected based on their capacity to provide influenza vaccination to the different age groups, their experience in performing EPSS, and their willingness to participate and vaccinate with the study vaccines. Healthcare professionals distributed paper safety report cards to individuals (or for participants aged <18 years, their parents or legal guardians) vaccinated with IIV3, IIV3-ID, or IIV4, and recorded the vaccination information for each participant daily using an electronic data capture system (eClinicalOS, Clinical Leader, PA, US). Participants were instructed to report any suspected ARs, especially those occurring in the first 7 days, by calling a dedicated local toll-free telephone number provided on the safety report card. ARs reported by each participant were collected using an Information Request Management System and were confirmed by a structured telephone interview as described previously.^8,9^ All events reported spontaneously by participants or healthcare professionals were considered as suspected ARs (i.e., vaccine-related) unless the participants specifically stated that they believed the events were unrelated or that a causal relationship could be excluded. No causality assessment was requested from the participants or healthcare professionals. The start of the EPSS coincided with the start of routine influenza vaccination for the NH influenza season 2017/18 by the selected study sites. The EPSS ended when 1000 safety report cards per vaccine had been distributed (+ 2 weeks for patient reporting) or 2 months after the first vaccinations (including 6 weeks for safety report card distribution + 2 weeks for patient reporting), whichever came first.

### Population size

The current interim guidance on EPSS for seasonal influenza vaccines in the EU requires the system to be able to detect ARs normally expected to be common (i.e., with a frequency ≥ 1%).^2^ To provide a > 99% probability of collecting ≥ 1 report of a given common AR, 1000 safety report cards per vaccine were targeted for distribution.

### Statistical analysis

For each vaccine, the rates of ARs and AEIs at the end of the surveillance period were calculated as the number of participants who reported ARs or AEIs and the total number of ARs or AEIs reported as numerators and the number of safety report cards distributed as the denominator. ARs were coded with Medical Dictionary for Regulatory Activities (MedDRA) terminology (version 20.1). AEIs were as listed in the PRAC guidance.^2^ Two-sided 95% confidence intervals (CIs) were calculated as described previously^8,9^ using SAS® version 9.4 (SAS Institute, Cary, NC, USA). For IIV3 and IIV3-ID, comparisons assessed if the current AR rates were greater or not than the upper limit of the 95% CI for those reported in the NH influenza season 2016/17. For IIV4, frequency categories were determined from the rates of ARs (very common, ≥10%; common, ≥1% to <10%; uncommon, ≥0.1% to <1%; rare, ≥0.01% to <0.1%; very rare, <0.01%) and compared with the documented frequency categories in the SmPC. Analyses were descriptive, and no confirmatory hypothesis testing was performed.
