# Supplementary material for: Enhanced passive safety surveillance of three marketed influenza vaccines in the UK and the Republic of Ireland during the 2017/18 season
Source: Hum Vaccin Immunother. 2019 Mar 27;15(9):2154–8. doi: 10.1080/21645515.2019.1581538 (PMC6773376; doi:10.1080/21645515.2019.1581538)
Supplement: Supplemental Material [file khvi-15-09-1581538-s001.docx]

## Supplementary Materials

**Supplementary Table 1: Severity of adverse events of interest (AEIs) for IIV3-ID, IIV3, and IIV4 during the Northern Hemisphere 2017/18 influenza season**

|  | **Total AEIs** | **AEI severity, n (%)** | | | |
| --- | --- | --- | --- | --- | --- |
| **Vaccine** |  | **Mild** | **Moderate** | **Severe** | **Unknown** |
| IIV3-ID | 37 | 18 (48.6) | 10 (27.0) | 7 (18.9) | 2 (5.4) |
| IIV3 | 17 | 11 (64.7) | 2 (11.8) | 1 (5.9) | 3 (17.6) |
| IIV4 | 25 | 9 (36.0) | 8 (32.0) | 6 (24.0) | 2 (8.0) |

Abbreviations: IIV3, trivalent split-virion inactivated influenza vaccine; IIV3-ID, intradermally administered trivalent split-virion inactivated influenza vaccine; IIV4, quadrivalent split-virion inactivated influenza vaccine
